# Identification of subclusters and prognostic genes based on glycolysis/gluconeogenesis in hepatocellular carcinoma

**DOI:** 10.3389/fimmu.2023.1232390

**Published:** 2023-10-10

**Authors:** Dan Chen, Ayinuer Aierken, Hui Li, Ruihua Chen, Lei Ren, Kai Wang

**Affiliations:** ^1^ School of Public Health, Xinjiang Medical University, Urumqi, China; ^2^ Department of Hepatobiliary Hydatid Disease, the First Affiliated Hospital of Xinjiang Medical University, Urumqi, China; ^3^ Central Laboratory, Xinjiang Medical University, Urumqi, China; ^4^ Center of Animal Experiments, Xinjiang Medical University, Urumqi, China; ^5^ Department of Burns, the First Affiliated Hospital of Xinjiang Medical University, Urumqi, China; ^6^ Department of Medical Engineering and Technology, Xinjiang Medical University, Urumqi, China

**Keywords:** hepatocellular carcinoma, glycolysis/gluconeogenesis, consensus clustering, overall survival, immune microenvironments

## Abstract

**Background:**

This study aimed to examine glycolysis/gluconeogenesis-related genes in hepatocellular carcinoma (HCC) and evaluate their potential roles in HCC progression and immunotherapy response.

**Methods:**

Data analyzed in this study were collected from GSE14520, GSE76427, GSE174570, The Cancer Genome Atlas (TCGA), PXD006512, and GSE149614 datasets, metabolic pathways were collected from MSigDB database. Differentially expressed genes (DEGs) were identified between HCC and controls. Differentially expressed glycolysis/gluconeogenesis-related genes (candidate genes) were obtained and consensus clustering was performed based on the expression of candidate genes. Bioinformatics analysis was used to evaluate candidate genes and screen prognostic genes. Finally, the key results were tested in HCC patients.

**Results:**

Thirteen differentially expressed glycolysis/gluconeogenesis-related genes were validated in additional datasets. Consensus clustering analysis identified two distinct patient clusters (C1 and C2) with different prognoses and immune microenvironments. Immune score and tumor purity were significantly higher in C1 than in C2, and CD4+ memory activated T cell, Tfh, Tregs, and macrophage M0 were higher infiltrated in HCC and C1 group. The study also identified five intersecting DEGs from candidate genes in TCGA, GSE14520, and GSE141198 as prognostic genes, which had a protective role in HCC patient prognosis. Compared with the control group, the prognostic genes all showed decreased expression in HCC patients in RT-qPCR and Western blot analyses. Flow cytometry verified the abnormal infiltration level of immune cells in HCC patients.

**Conclusion:**

Results showed that glycolysis/gluconeogenesis-related genes were associated with patient prognosis, immune microenvironment, and response to immunotherapy in HCC. It suggests that the model based on five prognostic genes may valuable for predicting the prognosis and immunotherapy response of HCC patients.

## Introduction

Liver cancer has increased in incidence worldwide, ranking sixth for incidence and fourth for mortality among all cancers (1). It is estimated that more than one million patients will be diagnosed with liver cancer each year by 2025 (2). In liver cancer, hepatocellular carcinoma (HCC) accounts for approximately 90% of primary liver tumors and is the most common primary tumor of the liver (3). Hepatitis B virus (HBV), hepatitis C virus (HCV), smoking, alcohol abuse, liver disease and liver injury are all risk factors for HCC (4). HCC patients have a poor prognosis and show an increasing trend worldwide. Due to late diagnosis, resistance to chemotherapy, frequent recurrence and metastasis, the 5-year overall survival (OS) rate of HCC patients has not significantly improved (5). The 5-year OS rate for early-stage HCC is more than 70%, while the median OS of patients with advanced HCC is 1-1.5 years (6). Therefore, it is imperative to screen and identify effective diagnostic and therapeutic strategies for HCC to improve the prognosis of this malignancy.

Recently, new studies have found that metabolic reprogramming may be another hallmark of cancer, contributing to the malignant biological properties of cancer (7). In the presence of oxygen, tumor cells exhibit high levels of glycolysis that provide energy for the metabolic activity of the cell, known as aerobic glycolysis or the Warburg effect (8). High levels of aerobic glycolysis, accompanied by massive glucose consumption and massive lactate production, confer proliferation, invasion and drug resistance advantages in tumor cells (9). Targeting enzymes related to glycolysis in HCC may be a selective therapeutic strategy (10). In addition, gluconeogenesis has a mechanism of action to inhibit glycolysis and block the progression of HCC (11). Although there have been studies constructing prognostic models for predicting survival (12, 13), it is still necessary to establish prognostic models related to glucose metabolism.

HCC has a multilayered heterogeneity that has been studied for many years with the aim of individualizing treatment of patients (14). Several studies have developed predictive models combining patient characteristics and biomarkers for HCC surveillance and early detection (15, 16). Cancer progression is not only controlled by cancer cells but also influenced by the tumor microenvironment (TME) formed by surrounding nonmalignant tumor cells. In recent years, studies have highlighted that glycolysis influences tumor growth and immune escape (17). Therefore, the identification of patient stratification and biomarkers from the perspective of the immune microenvironment and glycolysis is of great significance to improve the survival of HCC patients.

**Figure 1:**
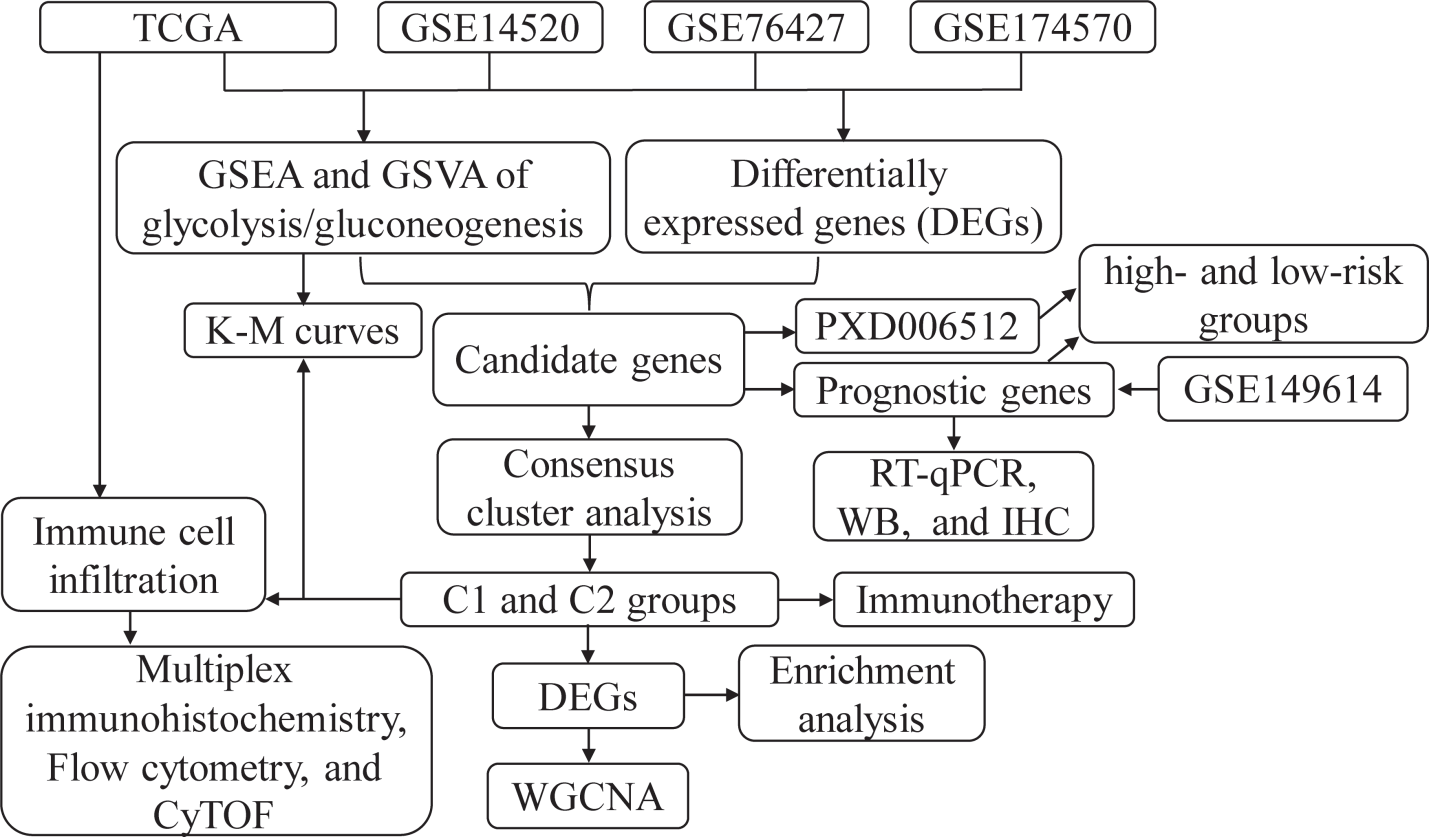
The flowchart of this study. CyTOF, Single‐cell‐scaled time‐of‐flight; GSEA, Gene Set Enrichment Analysis; GSVA, Gene set variation analysis; IHC, immunohistochemical; K-M, Kaplan-Meier; RT-qPCR, Real-time quantitative polymerase chain reaction; TCGA, The Cancer Genome Atlas; WB, Western blot; WGCNA, Weighted gene co-expression network analysis.

Analytical approaches through multi omics can comprehensively assess phenotypic heterogeneity in tumor samples, playing an important role in tumor marker screening and mechanistic studies (18, 19). In this study, we explored the important roles of metabolic reprogramming related genes in HCC patient stratification and prognosis based on high-throughput sequencing data. Further evaluation of the immune status of patients to evaluate the correlation of glycolysis and immune environment may help to reveal the pathogenesis and potential therapeutic avenues for HCC. This study suggests that our constructed glycolysis related multi prognostic model is an important component for HCC personalized therapy. The flowchart is shown in **Figure 1**.

## Materials and methods

### Data collection and differential analysis

Expression profile and survival data in the GSE14520 dataset were obtained from tissue samples of 247 HCC patients and 239 normal controls (NC) (20). Expression profile in the GSE76427 dataset were obtained from 115 primary tumors tissues and 52 adjacent non-tumor tissues of HCC patients (21). Expression profile in the GSE174570 dataset were obtained from 57 paired tumors tissues and adjacent non-tumor tissues of HCC patients (22). Expression profile and survival data in the GSE141198 dataset were obtained from tumors tissues of 148 HCC patients (23). In addition, expression, survival and somatic mutation data of 373 HCC patients and 50 normal controls were obtained from The Cancer Genome Atlas (TCGA) database (https://portal.gdc.cancer.gov/). Genes in four metabolic pathways (citrate cycle TCA cycle, fatty acid metabolism, glycerolipid metabolism, and glycolysis/gluconeogenesis) were collected from Molecular Signatures Database (MSigDB) (24). PXD006512 was collected from PRIDE database (25), including proteomic data with 124 paired tumors tissues and adjacent non-tumor tissues of HCC patients. Single-cell RNA sequencing (scRNA-seq) data were collected from GSE149614 for 10 HCC patients with tumor and non-tumor liver (26).

Differential analysis between HCC and controls were assessed using limma package in R (27) for GSE14520, GSE76427, GSE174570, and PXD006512, and using DEseq2 package in R (28) for TCGA, then differentially expressed genes (DEGs) and differentially expressed proteins (DEPs) were obtained with |logFC (log fold change)|> 1 and *P* < 0.05.

### Identification of metabolic pathways with prognostic significance

Gene Set Enrichment Analysis (GSEA) gene set (29) was used to evaluate the activation of four metabolic pathways in HCC. The scores of four metabolic pathways were calculated separately with gene set variation analysis (GSVA) (30), and the effect of the median score on patient OS was analyzed by Kaplan-Meier (K-M) curves. Subsequently, DEGs in metabolic pathways with prognostic significance were screened out as candidate genes.

### Consensus cluster analysis

Candidate genes were used to perform consensus cluster analysis using ConsensusClusterPlus package in R (31). HCC samples were therefore clustered as different clusters. The K-M curves were used to analyze and compare the OS of different clusters. DEGs between clusters in GSE14520, GSE141198, and TCGA were obtained with *P* < 0.05. Somatic mutation was calculated to evaluate the tumor mutation burden (TMB) in different clusters using Maftools (32). The stromal score, immune score, ESTIMATE score, tumor purity, and glycolysis/gluconeogenesis in TCGA were assessed between different clusters using GSVA.

### Co-expression and enrichment analysis

Weighted gene co-expression network analysis (WGCNA) was performed to construct co-expression networks in TCGA for intersecting DEGs between clusters in GSE14520, GSE141198, and TCGA using WGCNA package in R (33). Correlations between pairs of genes were first calculated using gene expression profiles and transformed into adjacency matrices. Then the optimal β was set such that the connections between genes in the network obeyed a scale-free network distribution, and the adjacency matrix was transformed into a topological overlap matrix. Hierarchical clustering trees were subsequently constructed, with different branches (colors) representing different modules. Correlations between modules and clinical trait were calculated by Pearson correlation.

Enrichment analysis was used to identify the Gene Ontology (GO) analysis, and Kyoto Encyclopedia of Genes and Genomes (KEGG) in which module genes were involved using clusterProfiler package in R (34). Significant pathways were determined by *P* < 0.05.

### Assessment of immune cell infiltration

CIBERSORT (35) was used to determine the immune cell infiltration based on the gene expression data in TCGA. The proportion of infiltrating immune cells was estimated by LM22 signal between HCC and control or between different clusters. Tumor Immune Dysfunction and Exclusion (TIDE; http://tide.dfci.harvard.edu/) was utilized to predict the responsiveness of samples in different clusters to immunotherapy.

### Identification of molecular subtypes

Univariate Cox regression was used to analyze the prognostic role of candidate genes in GSE14520, GSE141198, and TCGA. Forest plot was plotted with hazard ratio (HR) and 95% confidence interval (CI). Genes that significantly affected patients’ overall survival were identified as prognostic genes. Nomogram was generated using rms package in R. The calibration curves were established to illustrate the agreement between the nomogram-predicted and the observed probabilities of HCC. Then a risk score was calculated and the HCC samples in TCGA were divided into high- and low-risk groups according to the median risk score. OS in different groups was predicted by K-M curve. The receiver operating characteristic (ROC) curves was generated by survivalROC package in R to evaluate time-dependent OS of HCC patients.

### Validation in proteomic data

Multivariate Cox regression was used to analyze the prognostic role of candidate genes in protein level of PXD006512. Proteins that significantly affected patients’ OS were used to calculate a risk score. The HCC samples in PXD006512 were divided into high- and low-risk groups according to the median risk score. OS in different groups was predicted by K-M curve. The ROC curves were also generated by survival ROC package in R to evaluate time-dependent OS of HCC patients.

### Application of single-cell data

To filter out low-quality cells, cells with more than 8000 or fewer than 200 expressed genes were removed. Single cell sequencing data were normalized using a standardized data algorithm, and variable genes were filtered using the FindVariableFeatures function. Highly variable genes of top 2000 were selected to perform clustering analysis using unified manifold approximation and projection (UMAP). Marker genes for cell types were collected from known cell specific marker genes.

### Sample collection

A total of 30 paired tumors tissues and adjacent normal tissues from HCC patients were collected in the First Affiliated Hospital of Xinjiang Medical University. Peripheral blood samples of 10 HCC patients and 10 healthy volunteers were also collected in the First Affiliated Hospital of Xinjiang Medical University. Samples in this study were obtained with approval by Ethics Committee of the First Affiliated Hospital of Xinjiang Medical University (NO. K202304-20), and consent were obtained for all participants.

### Real-time quantitative polymerase chain reaction

Tumors tissues and normal tissues samples were used to extract total RNA using was TRIzol reagent (Invitrogen, CA, USA). Complementary DNA was obtained through reverse-transcribe using total RNA with PrimeScript™ RT reagent Kit (Takara, Dalian, China). Expression of target genes were detected using RT-qPCR with SYBR^®^ Premix Ex Taq™ II kit (Takara). β-actin is used as an internal reference gene to calculate the relative expression level of genes using 2^−ΔΔCt^ method. Primers used in this study is shown in **Table S1**.

### Western blot analysis

Tumors tissues and normal tissues samples were homogenated in RIPA lysis buffer with PMSF on ice. Proteins were extracted and then quantified with BCA protein assay kit (Beyotime, Shanghai, China). Then 20 µg proteins were separated on SDS–polyacrylamide gel electrophoresis and transferred onto polyvinylidene fluoride membranes. After blocking with 5% skim milk at room temperature for 2 h, membranes were incubated with primary antibodies (anti-ADH1B, anti-ALDOB, anti-ADH1A, anti-FBP1, anti-ADH6, and anti-β-actin; ABclonal Technology, Wuhan, China) at 4°C for overnight, respectively. Subsequently, membranes were incubated with HRP-linked secondary antibodies and detected with an ECL chemiluminescence kit (Beyotime). Quantification of proteins was performed by normalized to β-actin using ImageJ software.

### Immunohistochemical and multiplex immunohistochemistry staining

For immunohistochemical (IHC) staining, tissues were embedded in paraffin after fixed in 4% paraformaldehyde and sectioned at 4 μM. After antigen retrieval at high temperature, sections were incubated with sheep serum albumin for blocking antigen. Sections were then incubated with primary antibodies (anti-ADH1B, anti-ALDOB, anti-ADH1A, anti-FBP1, and anti-ADH6; ABclonal Technology) for overnight. Secondary antibody was applied for 30 min. After added the diaminobenzidine solution, sections were stained with hematoxylin. The images were visualized by XSP-C204 biomicroscope.

Additionally, sections were stained with anti-mouse CD14 monoclonal antibody (Proteintech, Wuhan, China) for 15 h, then incubated with FITC-conjugated goat anti-mouse IgG (Proteintech) for 1 h. After washing with solution for 4 times, sections were incubated with sheep serum albumin for blocking antigen. Then sections were stained with anti-rat FOXP3 polyclonal antibody (Absin, Shanghai, China) for 15 h, then incubated with Cy3-conjugated goat anti-rat IgG (Proteintech) for 1 h. Nuclei were counterstained with DAPI. The images were visualized by XSP-C204 biomicroscope.

### Flow cytometry assay

Peripheral blood samples of HCC patients and normal controls were collected and incubated with antibodies (BD Biosciences, CA, USA), including anti-CD14-ECD, anti-CD4-APC, anti-CD45RO-PE, anti-CD25-FITC, anti-CD127-APC, anti-CXCR5-PC5.5, and anti-CD68-PC7. After incubation for 18 min, cells were incubated with red blood cell lysate (BD Bioscience). After washing with PBS, cell population was gated and analyzed by BD FACSFortessa (BD Biosciences). Data were analyzed by Kaluza (v2.0).

### Data processing of mass cytometry

Single‐cell‐scaled time‐of‐flight (CyTOF) mass cytometry data was collected from Mendeley Database (https://doi.org/10.17632/jxsz3hdsyg.2) with accession numbers: CRA001276 (36). Which including thirteen groups of tumor (T), and normal (N) specimens from HCC patients. Cluster analysis was performed on CD45 positive cells using t-distributed stochastic neighbor embedding (tSNE), and subsequent manual gating was performed using FlowJo v10.5.3. Treg was identified using CD4+CD3+CD25+CD127low, monocyte was identified using CD3-CD16-CD4+HLA-DR+CD45RO+CD11a+CD49d+.

### Statistical analysis

R version 3.6.1 was used for bioinformatics analysis. GraphPad Prism 7.0 was used for statistical analysis and graphing. Data of experiment are expressed as mean ± SD for triplicate experiments. Statistical test was performed using Student’s t test. *P* < 0.05 was considered statistically significant.

## Results

### Prognostic significance of metabolic pathways

**Figure 2:**
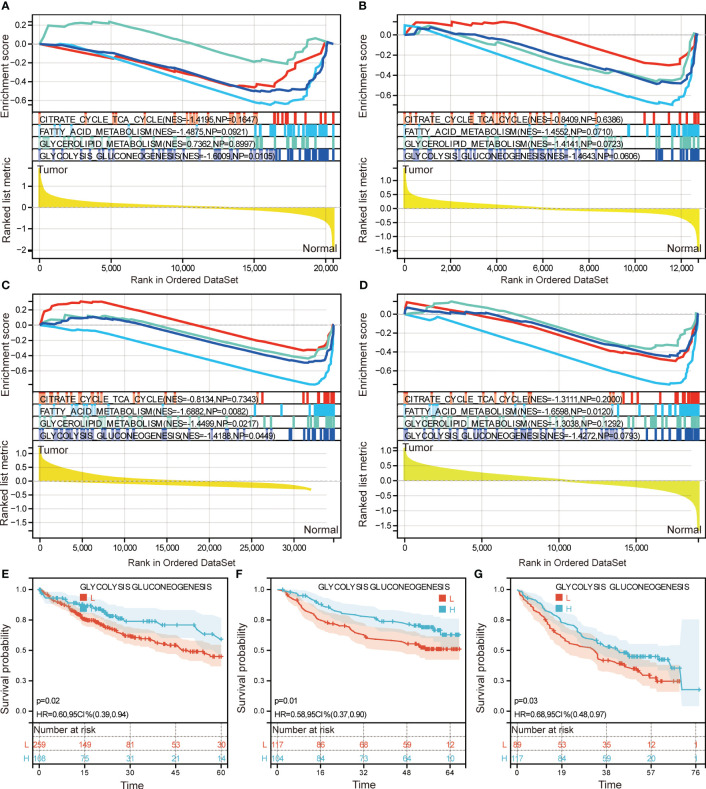
Identification of metabolic pathways with prognostic significance. Gene set enrichment analysis of citrate cycle TCA cycle, fatty acid metabolism, glycerolipid metabolism, and glycolysis/gluconeogenesis in TCGA (**A**) GSE14520 (**B**), GSE76427 (**C**), GSE174570 (**D**). Kaplan Meier survival analysis based on glycolysis/gluconeogenesis score in TCGA (**E**) GSE14520 (**F**), GSE141198 (**G**). H, high expression; L, low expression; HR, hazard ratio; CI, confidence interval.

To evaluate the metabolic pathways in HCC, GSEA was performed. The results showed that citrate cycle TCA cycle, fatty acid metabolism, glycerolipid metabolism, and glycolysis/gluconeogenesis were all activated in HCC in TCGA (**Figure 2A**), GSE14520 (**Figure 2B**), GSE76427 (**Figure 2C**), GSE174570 (**Figure 2D**). K-M curves showed that patients with high-score of glycolysis/gluconeogenesis had better OS compared to low-score of glycolysis/gluconeogenesis (**Figures 2E, F, G**). Unfortunately, the other three metabolic pathway scores did not significantly affect the OS of HCC patients.

### Differentially expressed glycolysis/gluconeogenesis‐related genes

**Figure 3:**
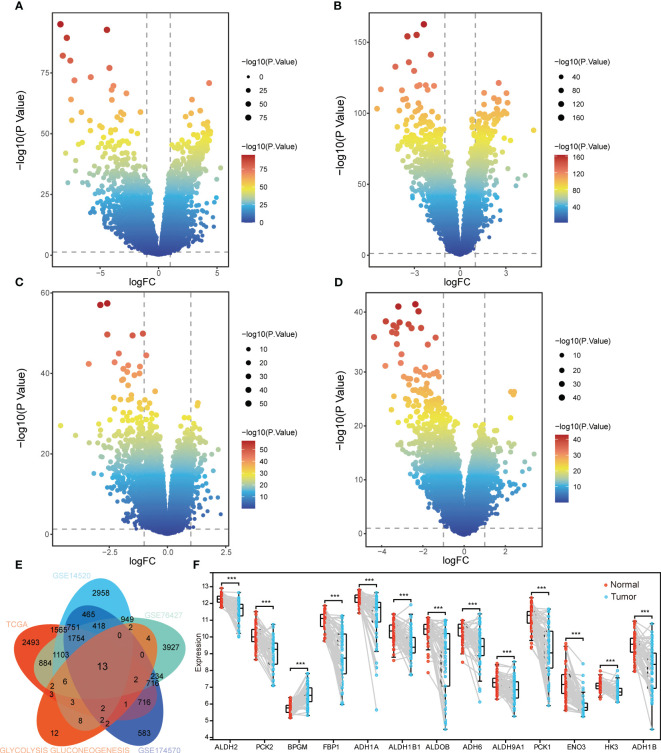
Identification of candidate genes associated with glycolysis/gluconeogenesis. Volcano plot of DEGs in TCGA (**A**), GSE14520 (**B**), GSE76427 (**C**), and GSE174570 (**D**). FC, fold change. **(E)** Intersection of DEGs in four datasets and glycolysis/gluconeogenesis‐related genes. TCGA, The Cancer Genome Atlas. **(F)** Differential expression of candidate genes in HCC and normal tissues of GSE174570. ***P < 0.001.

To identify differentially expressed glycolysis/gluconeogenesis‐related genes, the DEGs between HCC and controls were identified. A total of 2493 DEGs in TCGA (**Figure 3A**), 2958 DEGs in GSE14520 (**Figure 3B**), 3927 DEGs in GSE76427 (**Figure 3C**), and 583 DEGs in GSE174570 (**Figure 3D**). There were 62 glycolysis/gluconeogenesis‐related genes were obtained and intersection analysis revealed 13 differentially expressed glycolysis/gluconeogenesis‐related genes in HCC to be considered as candidate genes (**Figure 3E**). The expression of candidate genes in paired tumors tissues and adjacent non-tumor tissues of GSE174570 showed that BPGM was significantly higher expressed in HCC than in controls, and HK3, ENO3, ALDH1B1, ALDH9A1, ADH6, ADH1A, ADH1B, PCK1, ALDOB, FBP1, ALDH2, and PCK2 were significantly lower expressed in HCC (**Figure 3F**). Importantly, the aberrant expression of candidate genes was validated in additional data (**Figure S1**).

### Consensus clustering identified two clusters

**Figure 4:**
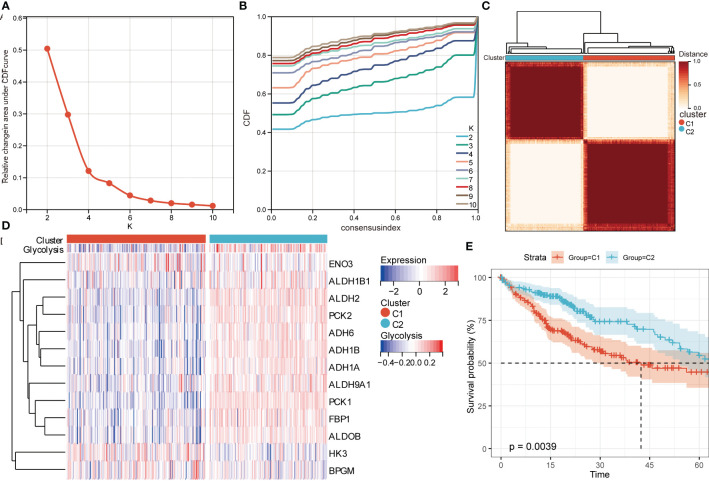
Identification of two HCC groups using consensus clustering analysis in TCGA based on candidate genes. **(A)** The relative change in area under cumulative distribution function (CDF) curve from k = 2 to 10. **(B)** The CDF curves from k = 2 to 10. **(C)** Heatmap of two clusters according to the consensus clustering matrix. **(D)** Expression heatmap of candidate genes in C1 and C2 groups. **(E)** Kaplan–Meier curves for OS of HCC patients in C1 and C2 groups. HR, hazard ratio; CI, confidence interval.

Based on the expression of 13 candidate genes, consensus cluster analysis was performed to discriminate HCC patients in TCGA. The greatest increase in the area under the cumulative distribution function (CDF) curves at k = 2 resulted in the best clustering (**Figures 4A–C**). Therefore, we obtained two clusters: C1 group and C2 group. The expression of 13 candidate genes in C1 and C2 was shown in **Figure 4D**. Patients in C1 group had a significantly worse OS than those in C2 group (**Figure 4E**). Interestingly, the same clustering results were also obtained in the GSE14520 and GSE141198 datasets, with patients in C1 group had a worse prognosis than those in C2 (**Figure S2**). Additionally, TP53 was found to be mutated most frequently in C1 and CTNNB1 in C2 among somatic mutations (**Figure S3**).

**Figure 5:**
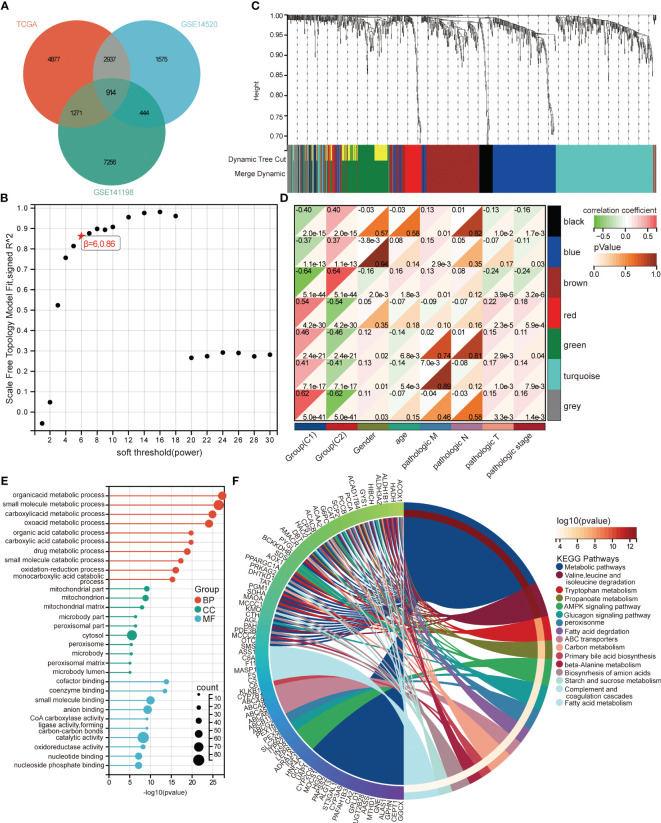
Co-expression network analysis in TCGA. **(A)** Intersection of differentially expressed genes between C1 and C2 groups in TCGA, GSE14520, and GSE141198 datasets. **(B)** Analysis of soft thresholding power (β) in WGCNA. **(C)** Clustering tree of seven co-expression modules. **(D)** Correlation between modules and clinical traits. Red represents positive correlation and green represents negative correlation. **(E)** GO terms enriched by brown module genes. BP, biological progression; CC, cellular composition; MF, molecular function. **(F)** KEGG pathways enriched by brown module genes.

Next, the differentially expressed genes between C1 and C2 groups were identified. There were 9999 differentially expressed genes in TCGA (**Figure S4A**), 5870 differentially expressed genes in GSE14520 (**Figure S4B**), 9885 differentially expressed genes in GSE141198 (**Figure S4C**). A total of 914 differentially expressed genes were the intersection of three datasets (**Figure 5A**). Network topology analysis of soft threshold power reveals β=6 was the optimal value to construct the co-expression network (**Figure 5B**). Then seven modules were obtained (**Figure 5C**). Correlation analysis showed that brown module was greatest positive correlation was with C2 and negative correlation with C1 (**Figure 5D**). The enrichment analysis found that brown module genes were mainly involved in organic acid metabolic process, small molecule metabolic process, and carboxylic acid metabolic process of biological processes (**Figure 5E**). In the KEGG pathways, metabolic pathways, valine, leucine and isoleucine degradation, and tryptophan metabolism were mainly enriched by brown module genes (**Figure 5F**).

### Immune and immunotherapy in two clusters

**Figure 6:**
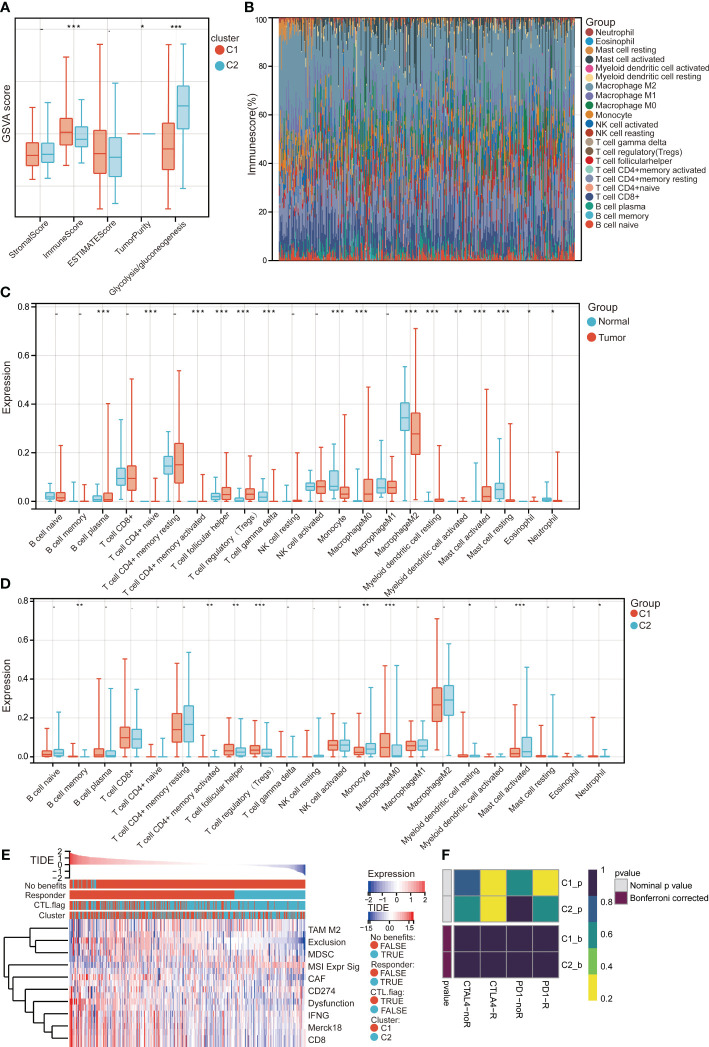
Immune cell infiltration and immunotherapy in C1 and C2 of TCGA. **(A)** Differences in estimate scores and glycolysis/gluconeogenesis for C1 and C2 groups. GSVA, gene set variation analysis. **(B)** Percentage abundances of different immune cells in each sample of HCC determined by CIBERSORT. **(C)** Differences in immune cell infiltration in HCC and controls. **(D)** Differences in immune cell infiltration in C1 and C2 groups. **(E)** Distribution of the TIDE value in C1 and C2 groups for immunotherapy response. CTL, cytotoxic T lymphocytes; TIDE, tumor immune dysfunction and exclusion; MDSC, myeloid-derived suppressor cells; CAF, cancer-associated fibroblasts; TAM, tumor-associated macrophages; MSI, microsatellite instability. **(F)** Therapeutic responses to anti-PD-1 and anti-CTLA-4 in C1 and C2 groups. *P<0.01, **P<0.01, ***P<0.001.

To investigate whether glycolysis/gluconeogenesis was associated with immune microenvironment, we explored the stromal score, immune score, ESTIMATE score, and tumor purity in C1 and C2 in TCGA. Results showed that immune score, and tumor purity were significantly higher in C1 than C2, while glycolysis/gluconeogenesis was lower in C1 than C2 (**Figure 6A**). Then the abundances of immune cells in each sample were measured using the CIBERSORT (**Figure 6B**). Macrophage M2, and CD4+ memory resting T cells were the most abundance in HCC. By comparing immune cell infiltration between HCC and controls, we found that CD4+ naive T cells, CD4+ memory activated T cells, follicular helper T cells (Tfh), regulatory T cells (Tregs), macrophage M0, myeloid dendritic cell resting, myeloid dendritic cell activated, and mast cell activated were higher infiltrated in HCC, while plasma B cells, gamma delta T cells, monocyte, macrophage M2, mast cell resting, eosinophil, and neutrophil were lower infiltrated in HCC (**Figure 6C**). Besides, memory B cell, CD4+ memory activated T cell, Tfh, Tregs, macrophage M0, myeloid dendritic cell resting, and neutrophil were higher infiltrated in C1, while monocyte, and mast cell activated were lower infiltrated in C1 (**Figure 6D**). CD4+ memory activated T cell, Tfh, Tregs, and macrophage M0 were all higher infiltration in HCC and C1 group.

Furthermore, differences in effects for patients in C1 and C2 receiving checkpoint inhibitors were predicted and found that the C1 group had a higher proportion of potential responders than the C2 group (**Figure 6E**). The responses to immunotherapies of receiving anti-PD-1 or anti-CTLA-4 were compared in C1 and C2 with the SubMap analysis (**Figure 6F**). It was found that HCC patients in C1 may be more sensitive for responses to anti-PD-1 and anti-CTLA-4.

### Identification of prognostic genes based on candidate genes

**Figure 7:**
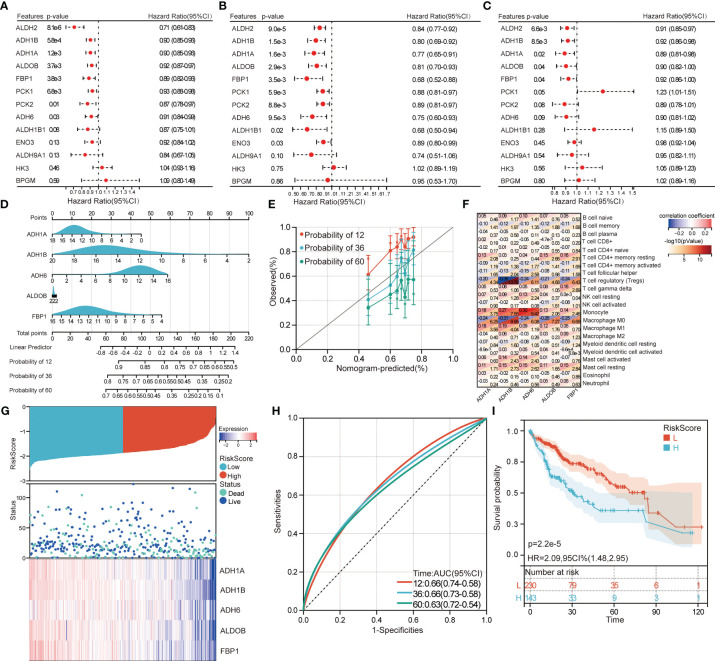
Identification of prognostic genes and construction of molecular subtypes. Univariate Cox regression analyzed prognostic role of candidate genes for HCC patients in TCGA (**A**), GSE14520 (**B**), and GSE141198 (**C**). **(D)** Nomograms for predicting overall survival in patients with HCC. **(E)** Calibration curves for predicting overall survival of the nomogram. **(F)** Correlations between immune cells and prognostic genes. Red represents positive correlation and blue represents negative correlation. **(G)** The HCC patients in TCGA were divided into high- and low-risk groups according to the median risk score. **(H)** ROC curves of median risk score for 12-, 36- and 60-month OS of HCC patients. AUC, area under the ROC curve. **(I)** Kaplan–Meier survival curves of high- and low-risk groups. H, high-risk group; L, low-risk group. HR, hazard ratio; CI, confidence interval.

Five intersecting DEGs were identified from candidate genes significantly affected OS in TCGA (**Figure 7A**), GSE14520 (**Figure 7B**), and GSE141198 (**Figure 7C**) by univariate Cox regression analyses. ADH1A, ADH1B, ADH6, ALDOB, and FBP1 as prognostic genes all had a protective role in HCC patient prognosis (HR<1). The nomogram was constructed for OS in HCC patients, and showed promising accuracy in predicting prognoses (**Figure 7D**). The calibration curves showed a robust calibration of nomogram (**Figure 7E**). The results of correlation analysis showed that prognostic genes and Treg or macrophage were negatively correlated, prognostic genes and monocyte was positively correlated (**Figure 7F**).

We then obtained two subtypes (high- and low-risk groups) in TCGA based on median risk score (**Figure 7G**). Prognostic genes were all lowly expressed in the high-risk group and highly expressed in the low-risk group. Area under the ROC curve (AUC) values of median risk score in 12-, 36- and 60-month were 0.66, 0.66 and 0.63, respectively (**Figure 7H**). Importantly, HCC patients in the high-risk group had a worse prognosis than those in the low-risk group (**Figure 7I**).

**Figure 8:**
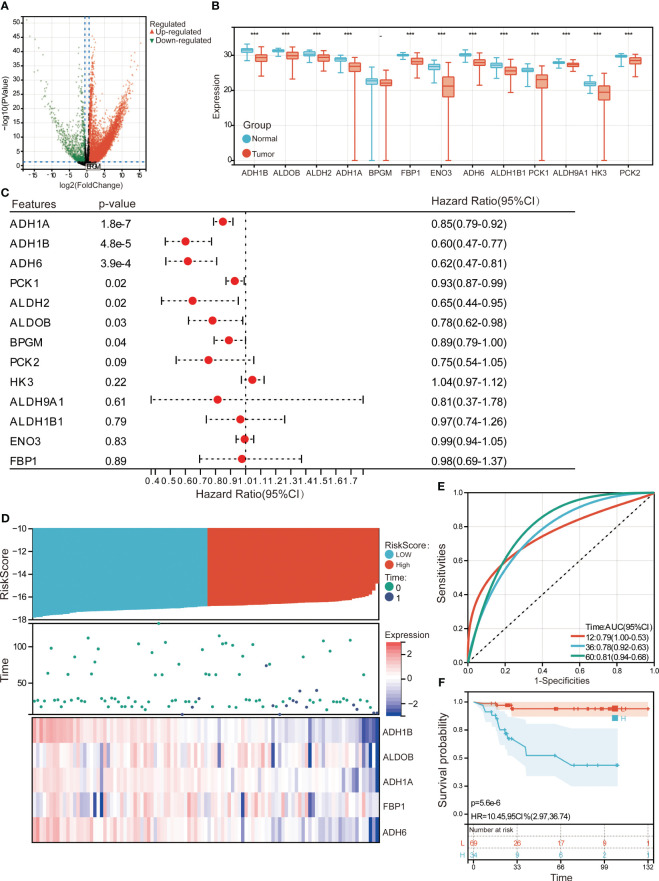
Evaluation of prognostic genes in PXD006512. **(A)** Volcano plot of differentially expressed proteins between HCC and controls. Red represents upregulation and green represents downregulation. **(B)** Protein expression levels of candidate genes. ***P<0.001. **(C)** Univariate Cox regression analysis of candidate genes for prognosis of HCC patients. **(D)** The HCC patients were divided into high- and low-risk groups according to the median risk score. **(E)** ROC curves of median risk score for 12-, 36- and 60-month OS of HCC patients. AUC, area under the ROC curve. **(F)** Kaplan–Meier survival curves of high- and low-risk groups. H, high-risk group; L, low-risk group. HR, hazard ratio; CI, confidence interval.

To determine the prognostic role of candidate genes in protein levels, the proteomic data in PXD006512 was analyzed. There were 5714 DEPs between HCC and controls (**Figure 8A**). Interestingly, the protein levels of the candidate genes were all lower expressed in HCC compared to controls (**Figure 8B**). Univariate Cox regulation analysis confirmed that ADH1A, ADH1B, ADH6, and ALDOB had protective effects on HCC (**Figure 8C**). A risk prognostic model was also established by median risk score prognostic genes to divide HCC samples into high - and low-risk groups (**Figure 8D**). Protein levels of prognostic genes were also lower expressed in high-risk group compared to low-risk group. AUC values of median risk score in 12-, 36- and 60-month were 0.79, 0.78 and 0.81, respectively (**Figure 8E**). HCC patients in the high-risk group had a worse prognosis than those in the low-risk group (**Figure 8F**).

### Integrated prognostic genes in single-cell level

**Figure 9:**
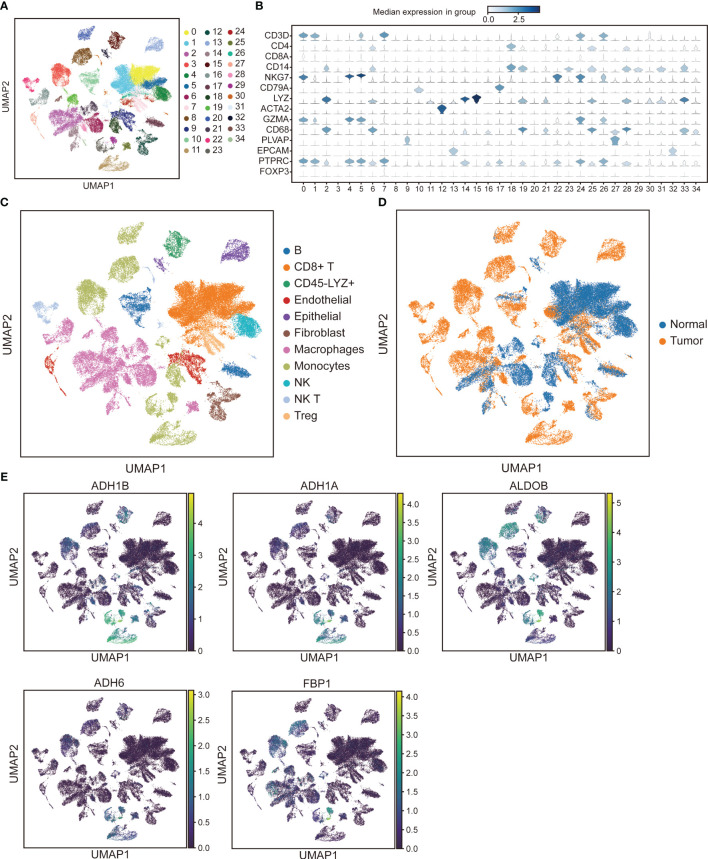
Single-cell analysis reveals major cell types and expression of prognostic genes. **(A)** Uniform manifold approximation and projection (UMAP) plots of different cell clusters. **(B)** Average expression and distribution of marker genes in all samples. **(C)** UMAP plots of annotated major cell types based on specific marker genes. NK, Nature killer. **(D)** UMAP clustering of HCC and normal samples. **(E)** UMAP plots of prognostic genes expression in cell types.

Unsupervised cluster analysis was performed 34 clusters were established through UMAP from cells of all samples (**Figure 9A**). According to specific marker genes, we identified the following 11 major cell types: B cells, CD8+ T cells, CD45-LYZ+ cells, endothelial, epithelial, fibroblast, macrophages, monocytes, natural killer (NK), NK T cells, and Treg (**Figures 9B, C**). Interestingly, NK T cells, and CD45-LYZ+ cells were more expressed in HCC samples, NK more expressed in normal samples (**Figure 9D**). ADH1A, ADH1B, ADH6, and ALDOB were mainly expressed in monocytes, FBP1 was mainly expressed in monocytes and macrophages (**Figure 9E**).

### Validation of prognostic genes and immune cells

**Figure 10:**
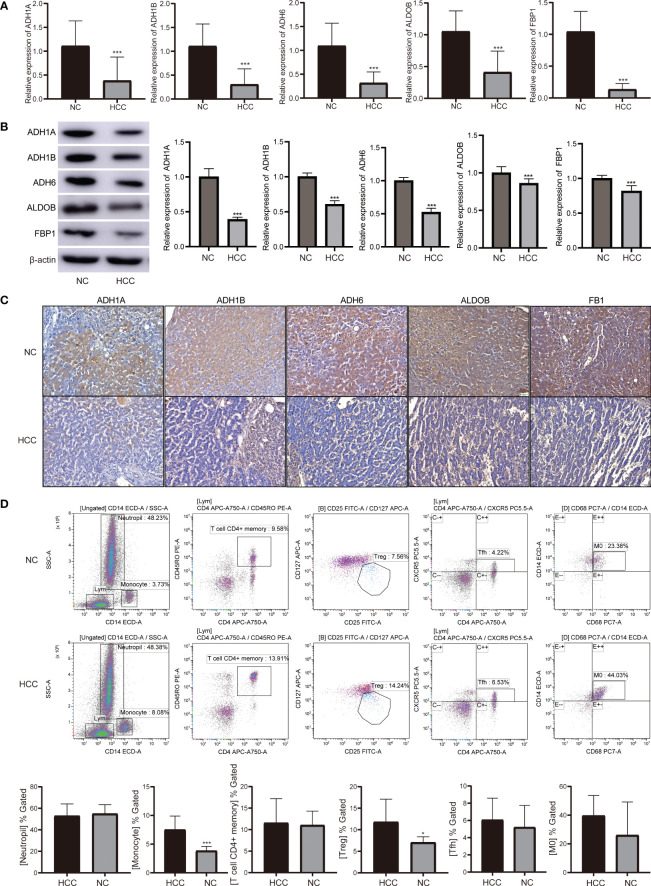
The levels of prognostic genes and immune cells were verified in HCC patients and normal controls. **(A)** The mRNA levels of prognostic genes in HCC and controls detected by RT-qPCR. **(B)** Protein expression of prognostic genes in HCC and controls detected by western blotting. **(C)** Protein expression of prognostic genes in HCC and controls detected by IHC staining. **(D)** The proportion of neutrophil, monocytes, CD4+ memory activated T cell, Tfh, Treg, and macrophages M0 detected by flow cytometry. ***P<0.001. HCC, hepatocellular carcinoma; NC, normal control; Tfh, follicular helper T cells; Treg, regulatory T cells.

RT-qPCR detection showed that decreased mRNA levels of ADH1B, ALDOB, ADH1A, ADH6, and FBP1 in HCC compared with controls (**Figure 10A**). The expression trend of prognostic genes was also decreased at the protein level in HCC (**Figure 10B**). We further confirmed that the expression of prognostic genes was lower in HCC than in the control group using IHC staining (**Figure 10C**).

**Figure 11:**
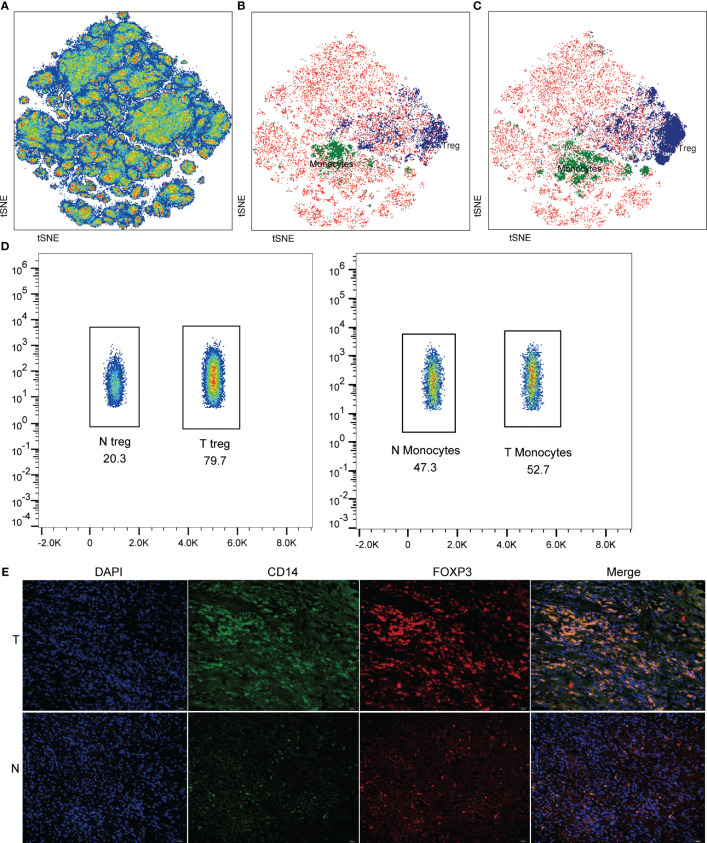
Detection of immune cells in HCC and normal groups. **(A)** tSNE plots showing CyTOF data from normal and tumor region. Identification of monocyte and Treg cells in normal **(B)** and tumor (**C**). **(D)** The proportion of monocyte and Treg cells in tumors and normal. **(E)** Representative images of immunohistochemistry staining for respective markers of monocyte and Treg cells in tumor (T) and normal (N).

The proportion of monocytes and Treg showed increased in HCC by flow cytometry detection (**Figure 10D**). Then, immune cells were visualized and analyzed based on CyTOF data (**Figure 11A**). According to the expression patterns of different immune cell surface markers (**Figure S5**), monocyte and Treg cells were identified significantly enriched in normal (**Figure 11B**), and tumor (**Figure 11C**). It is found that monocyte and Treg cells were increased from normal to tumor according to the cell density map (**Figure 11D**). Importantly, the multiplex immunohistochemistry staining was performed to detect the expression of marker protein of monocytes (CD14) and Treg (FOXP3). As shown in **Figure 11E**, the abundance of monocytes and Treg in tumor were higher than that in control group.

## Discussion

With the development of high-throughput sequencing technologies are being generated, which can be used to gain a better understanding of the underlying biology of HCC and to identify potential targets for diagnosis and therapy. In HCC, the process of glycolysis is often dysregulated and leads to increased glucose consumption and lactate production (37). Gluconeogenesis then exhibits anti-tumor effects in hepatocellular carcinoma (38). This study showed that several metabolic pathways were activated in HCC and may plays a significant role in HCC development and progression. The focus of this study is to discuss a study that identified differentially expressed glycolysis/gluconeogenesis-related genes in HCC and their association with immune microenvironment and patient prognosis.

The study found that patients with high scores for glycolysis/gluconeogenesis had better OS compared to those with low scores for glycolysis/gluconeogenesis. This suggests that glycolysis/gluconeogenesis may be a potential prognostic biomarker for HCC (39, 40). However, the scores for the other three metabolic pathways (citrate cycle/TCA cycle, fatty acid metabolism, and glycerolipid metabolism) did not significantly affect the OS of HCC patients. This suggests that these pathways may not be as strongly associated with HCC progression as glycolysis/gluconeogenesis.

Additionally, 13 candidate genes in HCC were all downregulated expression in HCC patients. Notably, two patient groups (C1 and C2) were identified based on the expression of candidate genes. The patients in C1 had a worse prognosis than those in C2. The different most frequently mutated genes in each cluster were also identified, which is the critical mechanism of tumorigenesis (41, 42). Patients in C2, characterized by lower expression of the 13 candidate genes, may have a higher probability of responding to checkpoint inhibitors compared to those in C1, which is consistent with the observation that patients in C2 had a better OS than those in C1. These results could have important implications for the development of personalized treatment strategies for HCC patients (43, 44).

The study also performed WGCNA to identify co-expression network for DEGs between C1 and C2. Correlation analysis revealed that the brown module had the greatest positive correlation with the C2 group and a negative correlation with the C1 group. Enrichment analysis on the brown module genes showed that they were mainly involved in metabolic pathways. Recently, many studies have found that the enzymes and metabolites of tryptophan metabolism are widely involved in the regulation of the immune system (45–47).

The role in metabolism on immunoregulation has attracted more attention. Many glycolysis related genes were found to be aberrantly expressed, and they play important roles in the development and recurrence of HCC (48). The expression of key enzymes involved in gluconeogenesis, is downregulated in HCC cells, leading to decreased glucose production (49). The accumulation of lactate, the main product of glycolysis, can affect tumor-related immune responses (50). Studies have shown that glycolysis-related gene signature can predict the survival and immune status of HCC (51). Then CIBERSORT was used to measure the abundance of immune cells in each sample and found several types of immune cells abnormally infiltrated in HCC and C1 group. Similar to HCC group, the immune level in C1 increases while the level of glycolysis decreases.

These findings suggest that glycolysis/gluconeogenesis may play a role in modulating the immune microenvironment in HCC (52). Specifically, the C1 group showed a more active immune response and higher infiltration of certain types of immune cells, which may be relevant for developing new therapeutic strategies targeting the immune system in HCC. Tregs are significantly elevated in the peripheral blood of HCC patients and represent an independent risk factor for prognosis (53). In addition, tumor associated neutrophils recruit macrophages and T regulatory cells that negatively regulate adaptive immunity, promoting progression through the CCL17 pathway (54, 55). With significantly increased Tfh and macrophages M0 in HCC, patients have a worse prognosis (56).

Cox regression analyses revealed ADH1A, ADH1B, ADH6, ALDOB, and FBP1 had protective role in HCC patient prognosis. Expression of five prognostic genes were confirmed by proteomic data. The correlation between these prognostic genes and immune cells showed and negatively correlated with Tregs or macrophages and positively correlated with monocytes. Next, two subtypes (high- and low-risk groups) were obtained based on the median risk score. The risk score model based on the expression levels of these genes could be useful in clinical practice for risk stratification and personalized treatment for HCC patients.

scRNA-seq helps elucidate the existence of tumor heterogeneity, which is common at the molecular and clinical levels in HCC (57). There were 11 major cell types in samples based on single-cell data. We also found that the prognostic genes ADH1A, ADH1B, ADH6, and ALDOB were mainly expressed in monocytes, while FBP1 was mainly expressed in both monocytes and macrophages. In HCC patients, higher ADH1A expression is associated with good survival and a lower invasive disease state (58, 59). Aberrant loss of ALDOB and upregulation of glycolysis in HCC tumor cells (60). FBP1 is a rate limiting enzyme in gluconeogenesis, which is downregulated in HCC patients and associated with poor prognosis (61). These findings suggest that different cell types may have different roles in the development and progression of HCC and may be affected differently by the expression of specific genes.

There are several limitations of these results that should be taken into account. The sample size of some datasets is relatively small, which may limit the statistical power and generalizability of the findings. We only focused on the metabolic and immunological features of HCC, and the clinical relevance and applicability of the findings need to be evaluated. The study did not investigate the impact of potential confounding factors such as age, sex, and comorbidities, which may affect the accuracy of the results. The study did not investigate the genetic and epigenetic mechanisms underlying the molecular and immunological features of HCC, and further studies are needed to elucidate these mechanisms.

## Conclusion

This study identified two distinct molecular subtypes (C1 and C2) associated with glycolysis/gluconeogenesis of HCC, which showed significant differences in gene expression patterns, immune microenvironment, and clinical outcomes. The study identified a novel five prognostic genes (ADH1A, ADH1B, ADH6, ALDOB, and FBP1) that were significantly associated with OS in HCC patients, they were mainly expressed in monocytes and macrophages. Overall, these findings highlight the importance of considering the molecular subtypes and immune microenvironment of HCC for developing personalized treatment strategies and improving patient outcomes.

## Data availability statement

The datasets presented in this study can be found in online repositories. The names of the repository/repositories and accession number(s) can be found in the article/**Supplementary Material**.

## Ethics statement

This study was approved by Ethics Committee of the First Affiliated Hospital of Xinjiang Medical University (NO. K202304-20). The studies were conducted in accordance with the local legislation and institutional requirements. The participants provided their written informed consent to participate in this study.

## Author contributions

DC conceived the study and designed the major experiments. AA performed experiments. HL contributed to Materials and methods. RC analyzed data. DC and LR wrote the manuscript. KW contributed to revisions of the manuscript. All authors contributed to the article and approved the submitted version.

## Supplementary material

Supplementary Figure 1 Expression heatmap of candidate genes in TCGA, GSE14520, GSE76427, and GSE174570 datasets.

Supplementary Figure 2 Identification of two HCC groups using consensus clustering analysis based on candidate genes. The relative change in area under cumulative distribution function (CDF) curve from k = 2 to 10 in GSE14520 **(A)** and GSE141198 **(E)**. The CDF curves from k = 2 to 10 in GSE14520 **(B)** and GSE141198 **(F)**. Heatmap of two clusters according to the consensus clustering matrix in GSE14520 **(C)** and GSE141198 **(G)**. Kaplan–Meier curves for OS of HCC patients in C1 and C2 groups in GSE14520 **(D)** and GSE141198 **(H)**. HR, hazard ratio; CI, confidence interval.

Supplementary Figure 3 Somatic mutations of samples in C1 and C2. **(A)** Characteristics of TMB for C1 samples in TCGA. **(B)** Characteristics of TMB for C2 samples in TCGA. TMB, tumor mutation burden.

Supplementary Figure 4 Identification differentially expressed genes between C1 and C2 groups. **(A)** Volcano plot of differentially expressed genes in TCGA. **(B)** Volcano plot of differentially expressed genes in GSE14520. **(C)** Volcano plot of differentially expressed genes in GSE141198. Red represents upregulated expressed genes and green represents downregulated expressed genes.

Supplementary Figure 5 tSNE plots showing expression of surface molecules in normal and tumor region.
