# Supplementary figures and images for: Identification of subclusters and prognostic genes based on glycolysis/gluconeogenesis in hepatocellular carcinoma
Source: Front Immunol. 2023 Oct 10;14:1232390. doi: 10.3389/fimmu.2023.1232390 (PMC10597634; doi:10.3389/fimmu.2023.1232390)

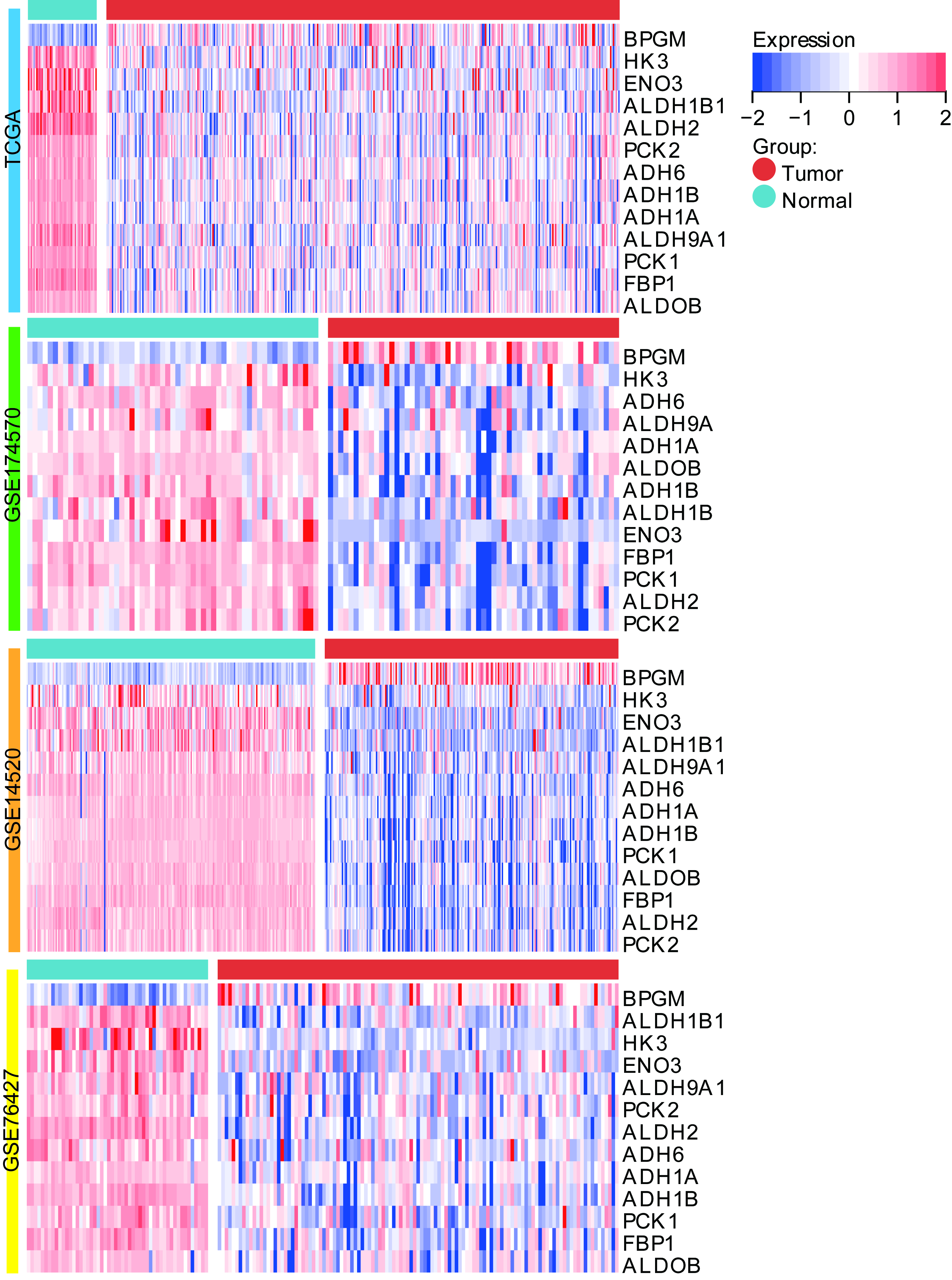

Supplement: Supplementary Figure 1 — Expression heatmap of candidate genes in TCGA, GSE14520, GSE76427, and GSE174570 datasets. [file Image_1.tif]

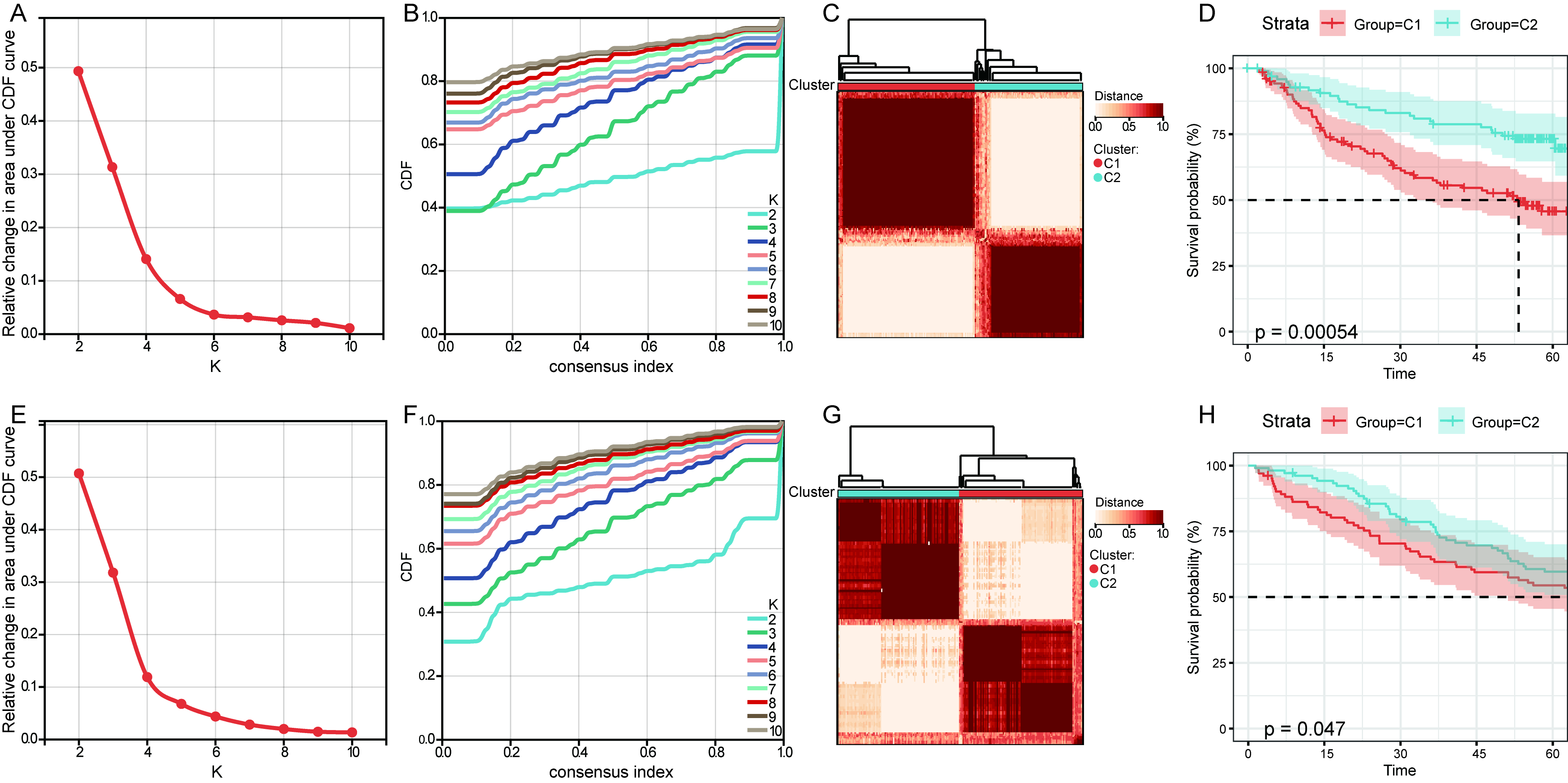

Supplement: Supplementary Figure 2 — Identification of two HCC groups using consensus clustering analysis based on candidate genes. The relative change in area under cumulative distribution function (CDF) curve from k = 2 to 10 in GSE14520 (A) and GSE141198 (E). The CDF curves from k = 2 to 10 in GSE14520 (B) and GSE141198 (F). Heatmap of two clusters according to the consensus clustering matrix in GSE14520 (C) and GSE141198 (G). Kaplan–Meier curves for OS of HCC patients in C1 and C2 groups in GSE14520 (D) and GSE141198 (H). HR, hazard ratio; CI, confidence interval. [file Image_2.tif]

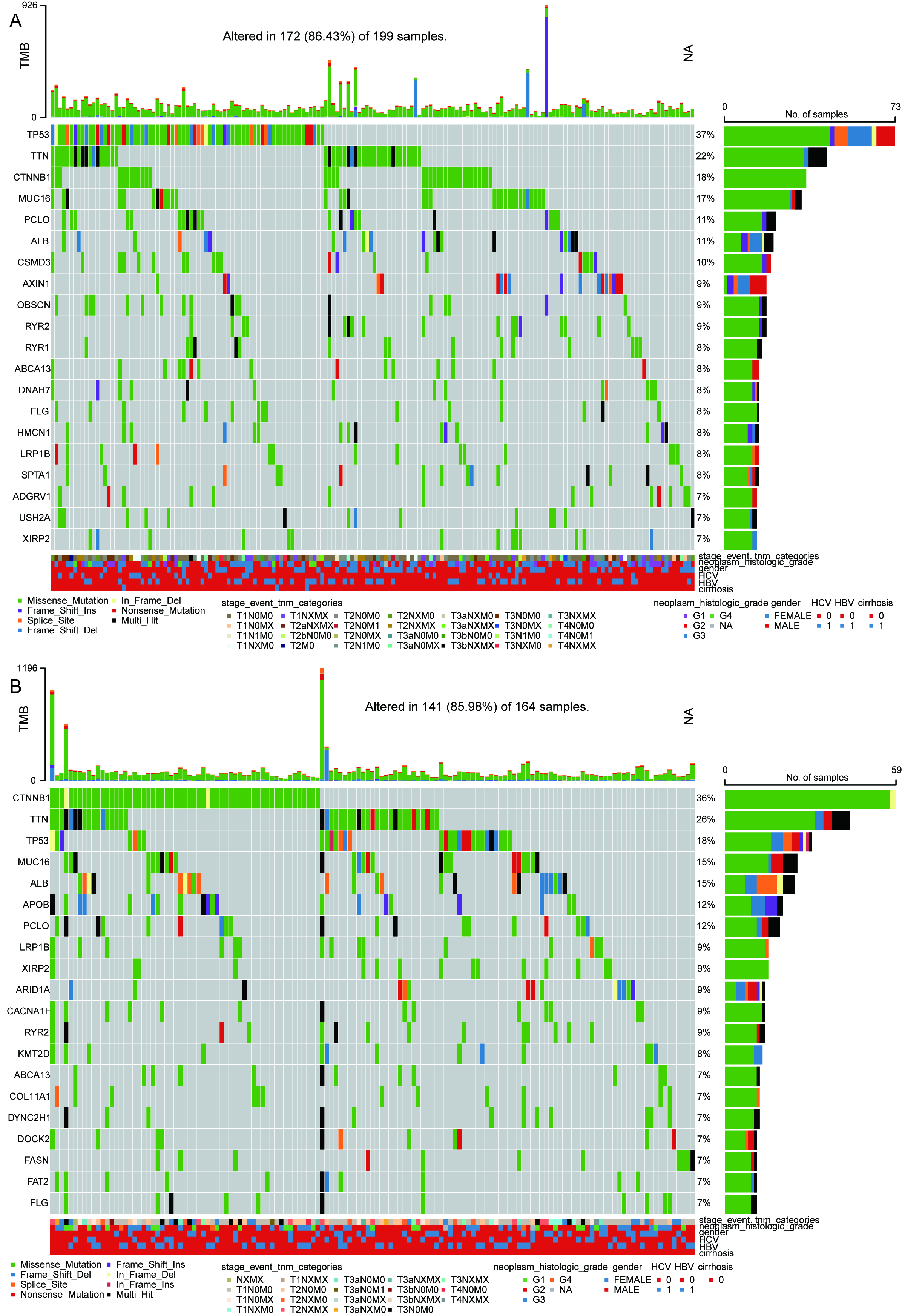

Supplement: Supplementary Figure 3 — Somatic mutations of samples in C1 and C2. (A) Characteristics of TMB for C1 samples in TCGA. (B) Characteristics of TMB for C2 samples in TCGA. TMB, tumor mutation burden. [file Image_3.tif]

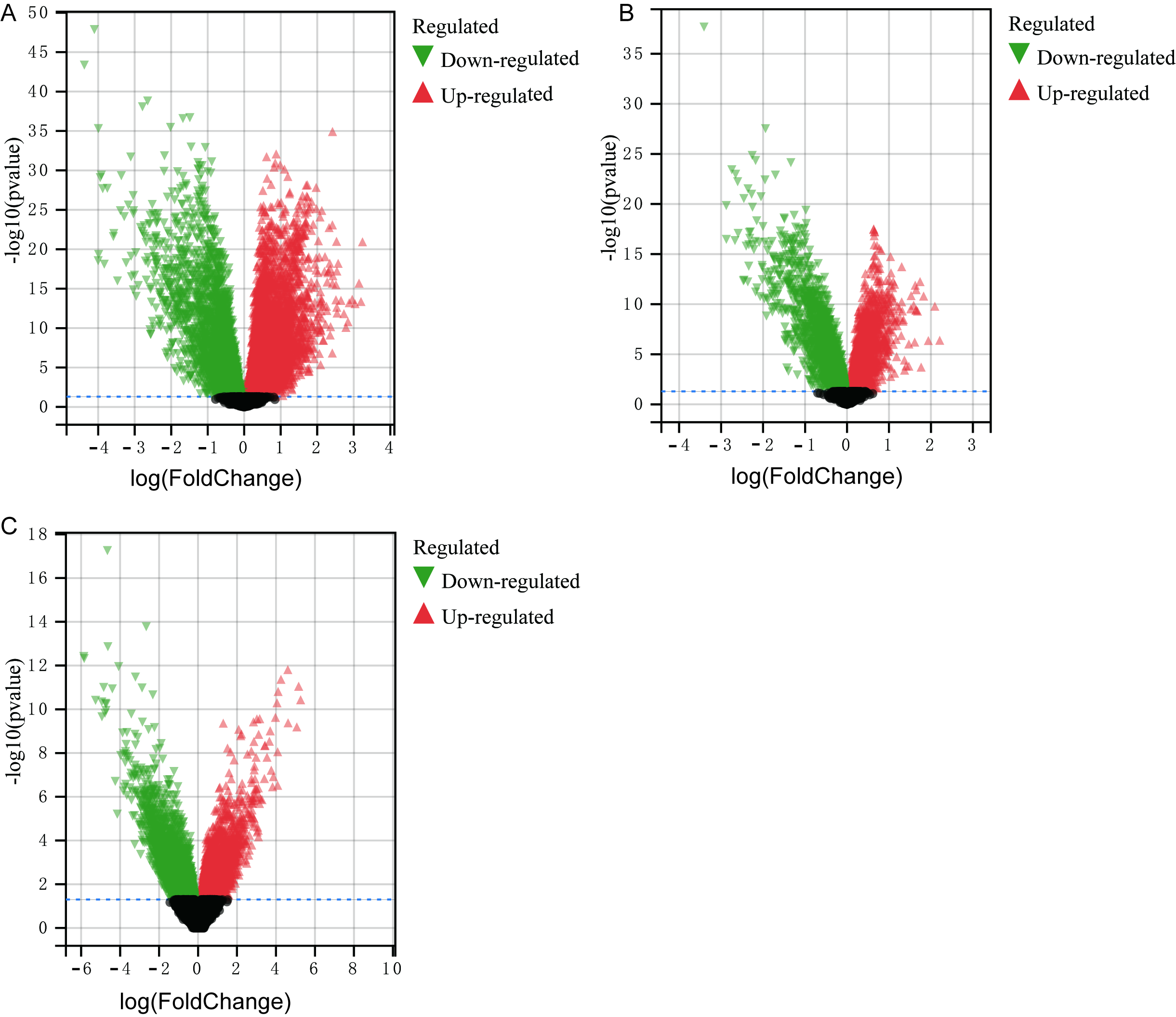

Supplement: Supplementary Figure 4 — Identification differentially expressed genes between C1 and C2 groups. (A) Volcano plot of differentially expressed genes in TCGA. (B) Volcano plot of differentially expressed genes in GSE14520. (C) Volcano plot of differentially expressed genes in GSE141198. Red represents upregulated expressed genes and green represents downregulated expressed genes. [file Image_4.tif]

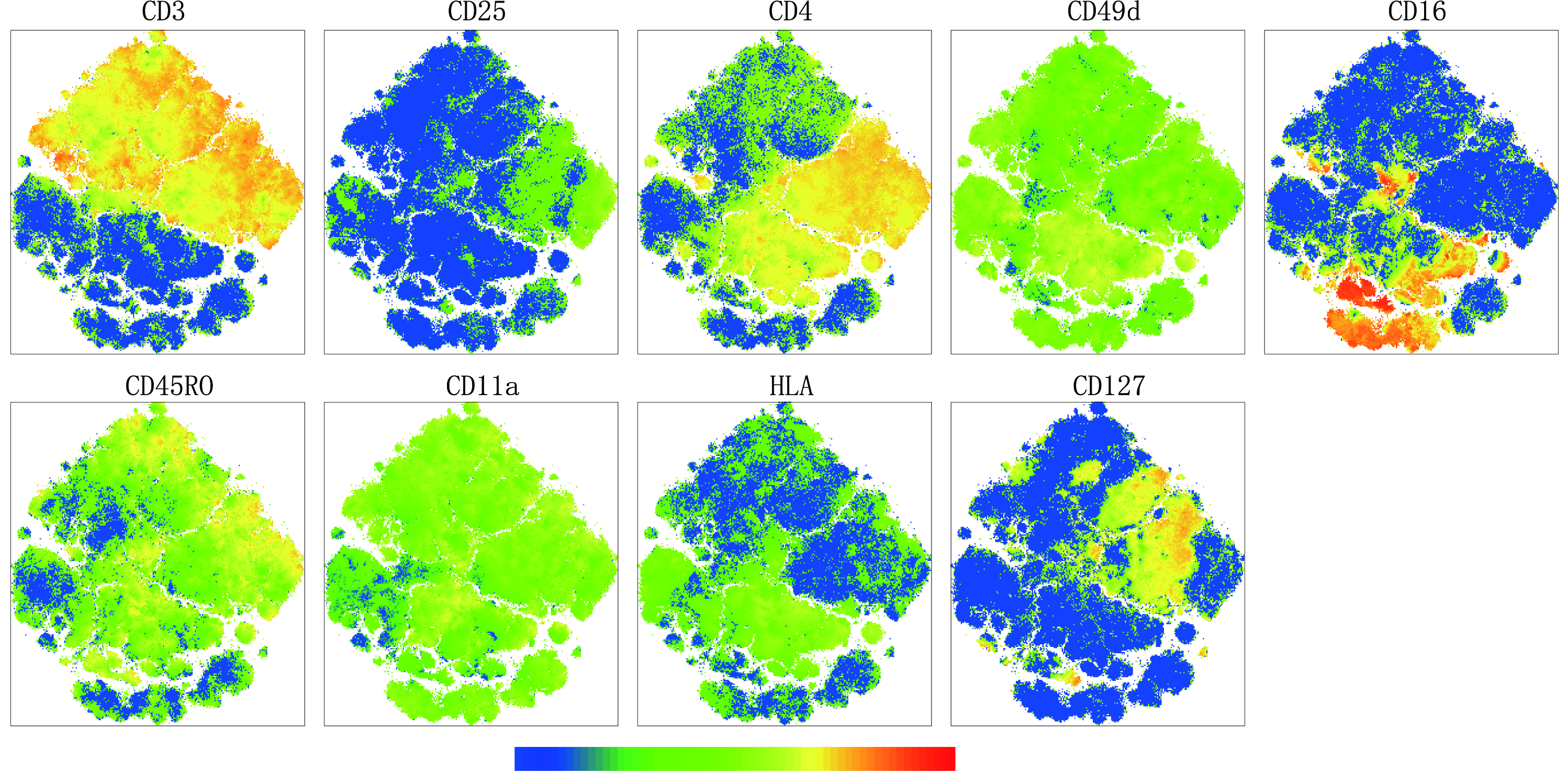

Supplement: Supplementary Figure 5 — tSNE plots showing expression of surface molecules in normal and tumor region. [file Image_5.tif]
