# Supplementary material for: Identification of subclusters and prognostic genes based on glycolysis/gluconeogenesis in hepatocellular carcinoma
Source: Front Immunol. 2023 Oct 10;14:1232390. doi: 10.3389/fimmu.2023.1232390 (PMC10597634; doi:10.3389/fimmu.2023.1232390)
Supplement: Supplementary file 6 [file Table_1.docx]

**Table S1. Sequences of primers (5’-3’) used for qRT-PCR.**

| Genes | | Sequences (5’-3’) |
| --- | --- | --- |
| ADH6 | FORWARD | GAGGAGTCGGCTTGTCTGTTGTC |
| ADH6 | REVERSE | GGCACTCAGTAGCACCCAATTCC |
| FBP1 | FORWARD | CATTCCCACAGACATTCACCAGAGG |
| FBP1 | REVERSE | GGCAGAGTGCTTCTCATACACCTTC |
| ALDOB | FORWARD | GGCGTGCTGTGCTGAGGATTG |
| ALDOB | REVERSE | CTGCTGACAGATGCTGGCGTAG |
| ADH1A | FORWARD | GGCTCTACCTGTGCTGTGTTTGG |
| ADH1A | REVERSE | GTTGATGTCCACCGCAATGATTCTG |
| ADH1B | FORWARD | GTGGCACAAGCGTCATCGTAGG |
| ADH1B | REVERSE | GCGTCCAGTCAGTAGCAGCATAG |
| β-actin | FORWARD | ACCAACTGGGACGACATGGAGAAA |
| β-actin | REVERSE | TAGCACAGCCTGGATAGCAACGTA |
